# Orthopaedic phenotyping of NGLY1 deficiency using an international, family-led disease registry

**DOI:** 10.1186/s13023-019-1131-4

**Published:** 2019-06-19

**Authors:** Eli M. Cahan, Steven L. Frick

**Affiliations:** 1 New York University School of Medicine, New York, NY 10010 USA; 2 Department of Pediatric Orthopaedics, Stanford University, 300 Pasteur Drive, R107, Palo Alto, CA 94305 USA

**Keywords:** NGLY1 deficiency, Orthopaedics, Natural history, Standard of care, Disease advocacy organizations, Evidence-based medicine, Disease registry

## Abstract

**Background:**

NGLY1 deficiency is a rare autosomal recessive disorder caused by loss in enzymatic function of NGLY1, a peptide *N*-glycanase that has been shown to play a role in endoplasmic reticulum associated degradation (ERAD). ERAD dysfunction has been implicated in other well-described proteinopathies, such as Alzheimer’s disease, Parkinson’s disease, and Huntington’s disease. The classical clinical tetrad includes developmental delay, hypolacrima, transiently elevated transaminases, and hyperkinetic movement disorders. The musculoskeletal system is also commonly affected, but the orthopaedic phenotype has been incompletely characterized. Best practices for orthopaedic clinical care have not been elucidated and considerable variability has resulted from this lack of evidence base. Our study surveyed patients enrolled in an international registry for NGLY1 deficiency in order to characterize the orthopaedic manifestations, sequelae, and management.

**Results:**

Our findings, encompassing the largest cohort for NGLY1 deficiency to date, detail levels of motor milestone achievement; physical exam findings; fracture rates/distribution; frequency of motor skill regression; non-pharmacologic and non-procedural interventions; pharmacologic therapies; and procedural interventions experienced by 29 participants. Regarding the orthopaedic phenotype, at time of survey response, we found that over 40% of patients experienced motor skill regression from their peak. Over 80% of patients had at least one orthopaedic diagnosis, and nearly two-thirds of the total had two or more. More than half of patients older than 6 years had sustained a fracture. Related to orthopaedic non-medical management, we found that 93 and 79% of patients had utilized physical therapy and non-operative orthoses, respectively. In turn, the vast majority took at least one medication (including for bone health and antispasmodic therapy). Finally, nearly half of patients had undergone an invasive procedure. Of those older than 6 years, two-thirds had one or more procedures. Stratification of these analyses by sex revealed distinctive differences in disease natural history and clinical management course.

**Conclusions:**

These findings describing the orthopaedic natural history and standard of care in patients with NGLY1 deficiency can facilitate diagnosis, inform prognosis, and guide treatment recommendations in an evidence-based manner. Furthermore, the methodology is notable for its partnership with a disease-specific advocacy organization and may be generalizable to other rare disease populations. This study fills a void in the existing literature for this population and this methodology offers a precedent upon which future studies for rare diseases can build.

## Introduction

*NGLY1* is a 70 kb gene composed of 12 exons and located on chromosome 3 that is highly conserved between eukaryotic species [1, 2]. The gene product, *N*-glycanase, is a conserved enzyme localized primarily to the cytoplasm that is involved in endoplasmic reticulum associated degradation (ERAD) [3]. Enzyme induced deglycosylation of misfolded glycoproteins located at the N-termini of polypeptides marks them for transport into the cytosol and proteasomal degradation [4, 5]. Additionally, NGLY1 is required for the transcriptional activity of NFE2L1, a protein with roles in regulating proteotoxic and oxidative stress. These roles are essential to (i) ensure appropriate function for proteins released to the cytosol and (ii) prevent toxic accumulation of malformed proteins within the cell [5, 6].

NGLY1 deficiency (OMIM 610661 and 615,273) is an autosomal recessive disorder that results in a complete or partial loss of *N*-glycanase activity in the cytosol [7]. There is in vitro evidence that such misfolded products of the endoplasmic reticulum (ER) aggregate within the cell and can cause deleterious effects on multiple subcellular organelles [7, 8]. In this proposed model of pathogenesis, affected organelles include the cytoplasm (which loses functional volume due to protein agglomeration) [8, 9]; endoplasmic reticulum (in which synthetic capability is diminished) [10]; mitochondria [11, 12]; and the proteasome (which incurs defects in functional subunits) [13].

The result may be unregulated cellular necrosis (rather than regulated cellular apoptosis or autophagy): a pathophysiology proposed to resemble other proteinopathies, such as Alzheimer’s disease (due to Tau accumulation) and Huntington’s disease (due to Htt protein accumulation) in animal models [8, 14].

Three cell types shown to have abnormalities in vitro include neurons, myocytes, and fibroblasts. Neurons have demonstrated susceptibility to proteinopathies due to high levels of protein production and low rates of cellular turnover [15]. Myocytes show considerable dependence on mitochondrial function and likewise have high levels of protein production [16]. Fibroblasts also have exhibited high levels of protein production, with extensive energy utilization as a result [8, 10].

Dysfunction and destruction of these cells leads to phenotypic effects across organ systems [17]. In humans, NGLY1 deficiency is a rare disorder with approximately 50 confirmed patients worldwide [17]. The disease classically presents with a clinical tetrad of developmental delay, hypolacrima, transiently elevated transaminases, and hyperkinetic movement disorders [17, 18].

Due to the rarity of the condition, there is a paucity of detailed phenotypic information available in the medical literature [17, 18]. Previously documented symptoms have focused on the neurologic, ophthalmologic, immunologic, and endocrine organ systems [7, 11, 15, 17–19]. Additionally, various efforts have identified specific, common musculoskeletal manifestations such as osteopenia, neuromuscular scoliosis, joint dysfunction, and muscular atrophy [17–19]. However, the orthopaedic phenotype and treatments undertaken for the musculoskeletal manifestations have been incompletely characterized.

Musculoskeletal findings that may have important clinical implications include increased risk of fractures, soft tissue injury, and spinal deformity. Patients frequently undergo procedural interventions, though the profile of interventions and their objective and subjective outcomes are not known. As a result, physicians are unable to provide evidence-based counseling and recommendations for these patients. Our study sought to elaborate the orthopedic manifestations, treatments, and outcomes experienced by patients with NGLY1 deficiency.

## Methods

The sample population consisted of 29 patients participating in a global registry initiated by the Grace Science Foundation in 2017 [20]. The Grace Science Foundation (https://gracescience.org/) is a nonprofit disease advocacy organization founded in 2014 to understand and treat NGLY1 deficiency.

All patient families participating in the registry between December 2017 and November 2018 answered a questionnaire addressing their child’s clinical symptoms, treatment course, and outcomes dating up until the time of enrollment. Within this questionnaire, a set of 23 orthopaedic specific questions was developed for evaluation of musculoskeletal manifestations and management therein (Supplement #1). IRB approval was obtained prior to enrollment of any patients.

Patient response data was compiled through secure information storage systems maintained by the Grace Science Foundation and shared securely with the investigator group. Quantitative analyses were conducted in Microsoft Excel and SPSS.

## Results

Our study cohort consisted of 29 patients, ranging in age from 9 months to 25 years. The average age was 9.4 years, with a median age of 9 years. 15 (51.7%) of the patients were female, and 14 (48.3%) male. The age of female respondents was 9.1 (+/− 13.3) years compared to 9.7 (+/− 10.3) years for males. Due to privacy concerns on account of the rarity of the disease, additional demographic details cannot be provided.

### Motor milestone achievement

**Fig. 1:**
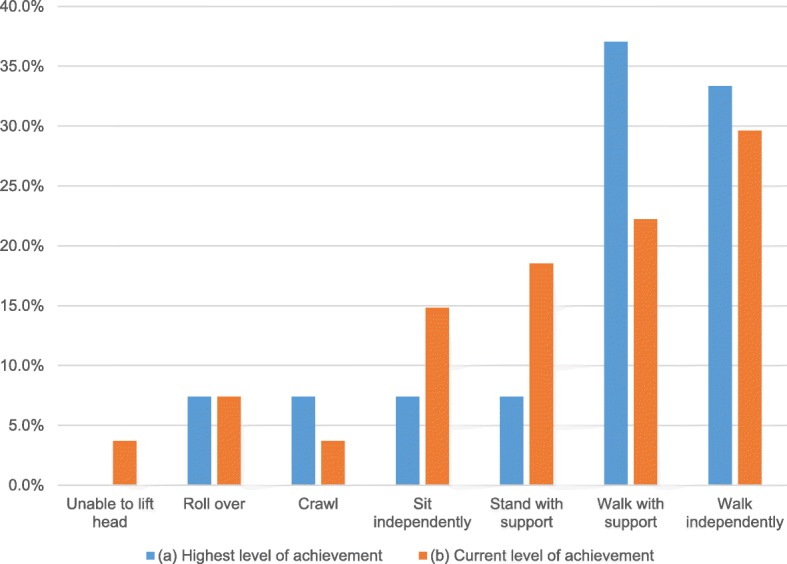
Level of motor milestone achievement by/at time of survey in patients older than 18 months

Achievement and regression of gross motor milestones provides a means for assessing the orthopedic implications of NGLY1 deficiency. Since independent walking is typically achieved between 9 and 18 months of age, the 27 patients older than 18 months in age at the time of the survey were assessed for milestone achievement. 9 (33.3%) had achieved independent walking (Gross Motor Function Classification System [GMFCS] Level I) at the time of survey, and another 10 (37.0%) were able to walk with brace and/or walker support (GMFCS II-III) [21]. Of the remaining 8 (40.8%), 2 (7.4%) each demonstrated peak motor achievement by: standing with support, sitting independently, crawling, or rolling over (Fig. 1a). The average age of highest milestone achievement was 35.6 months, and the median was 36 months. These patients were diagnosed with motor delay on average by the age of 4.8 months, with a median of 4 months.

11 of 27 (40.7%) experienced regression in motor skills. At the time of survey, of the 9 GMFCS I patients, 3 (33.3%) noted motor skills regression. 1 had regressed to GMFCS-V, and the other 2 noted gait abnormalities, imbalance, and rapid fatigue compared to earlier in life. Of the 10 GMFCS II-III patients, 5 (50%) had decreasing motor skills. 4 of these declined to GMFCS IV-V, and the fifth was situationally unable to walk with support (on inclines, up stairwells). Of the remaining 10 patients who had peaked at GMFCS IV, 3 (30%) had motor skill regression (Fig. 1b).

Comparing between sexes, the average age of onset for the index musculoskeletal abnormality was 14.4 vs. 13.5 months for females and males, respectively. Females tended to achieve their maximal milestones at an early age (29.3 vs. 42.3 months), and had motor skill delays detected later (5.6 vs. 3.9 months). Inability of antigravity head holding was a common presenting symptom in males, but was not seen in females (35.7% vs. 0%, respectively). In terms of maximal milestone achievement, females were more likely to have achieved independent walking at the time of survey than were males (40.0% vs. 21.4%). Females were also more likely to experience regression in motor skills from peak (40.0% vs. 35.7% in males), but the cohort that achieved independent walking was spared from regression compared to males (40.0% vs. 14.3% still able to walk independently at time of survey).

### Musculoskeletal manifestations

**Fig. 2:**
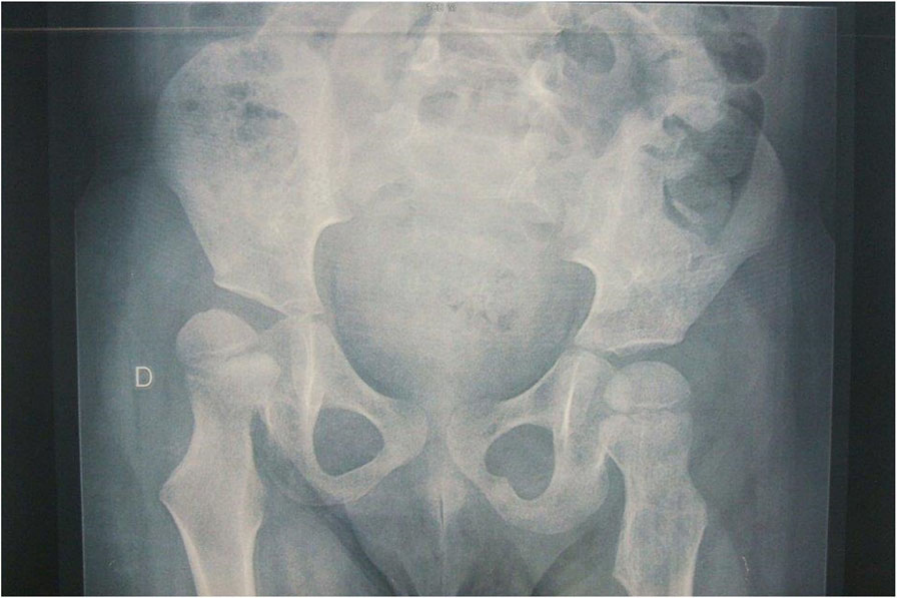
AP pelvis radiograph demonstrating classic signs of neuromuscular hip dysplasia including coxa valga, caput valgum, acetabular dysplasia, and subluxation of the hip joint

**Fig. 3:**
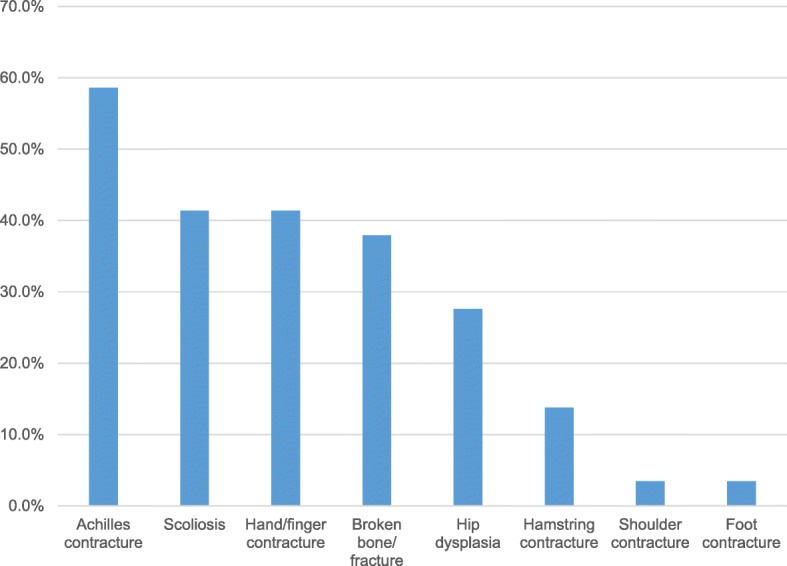
Prevalence of musculoskeletal manifestations in the study cohort

5 of 29 patients (17.2%) had exactly one orthopedic diagnosis, while nearly two thirds (19; 65.5%) of patients had greater than one. These findings varied from joint contractures to bone fractures to scoliosis to hip dysplasia (Fig. 2). Achilles contractures were the most common orthopaedic manifestation, noted in 17 patients (58.6%)(Fig. 3). Of the 11 (37.9%) patients to sustain bone fractures, 5 (17.2% of the total, and 45.4% of the fracture subset) experienced multiple fractures. The average number of fractures in patients with at least one was 2.4 (95% CI: 0.61–4.19 fractures). There was a wide distribution of fractures, with those in the lower extremity (excluding the foot/ankle) being the most common. The average age of those reporting one fracture or more was 12.4 years (95% CI: 8.1–16.7 years). Of the 18 patients older than 6 years, 10 (55.5%) had sustained a fracture (compared to baseline rates of 32.2–50.0% from 5 years old to young adulthood) [22].

Comparing sexes, males were more likely to report a medical history of scoliosis (57.1% vs. 16.0%), hip dysplasia (50.0% vs. 6.7%), Achilles tendon contractures (71.4% vs. 46.7%), upper extremity contractures (57.1% vs. 26.7%), and bone fractures (50.0% vs. 26.7%). Females were only more likely to report hamstring muscle contracture (20.0% vs. 7.1% in males). They also reported more bone fractures per patient when fractures had occurred (3 vs. 2 in males, on average). The three most common musculoskeletal manifestations reported in both sexes were Achilles tendon contractures, scoliosis, and upper extremity contractures. Females reported bone fractures at equal prevalence to scoliosis and upper extremity contractures (all three were reported in 16.0% of patients).

### Non-pharmacologic and non-procedural interventions for musculoskeletal disorders

Many patients underwent non-pharmacologic, non-surgical efforts for musculoskeletal diagnoses. 23 of 29 (79.3%) historically had utilized one or more orthoses: 21 (72.4%) for foot/ankle splinting, 9 (31.0%) for hand/wrist splinting, and 8 (27.6%) for spinal bracing. There were 53 reported instances of orthosis, 31 (58.5%) for foot/ankle splinting, 14 (26.4%) for hand/wrist splinting, and 8 (15.1%) for spinal bracing. 15 (65.2%) of those with orthoses; 51.7% of total) reported multiple orthoses at any point. Of the 25 patients older than 2 years old at time of survey, 23 (92%) reported use of an orthosis at any point. 2 patients reported the use of additional orthopedic support devices, such as cushions or orthotics.

**Fig. 4:**
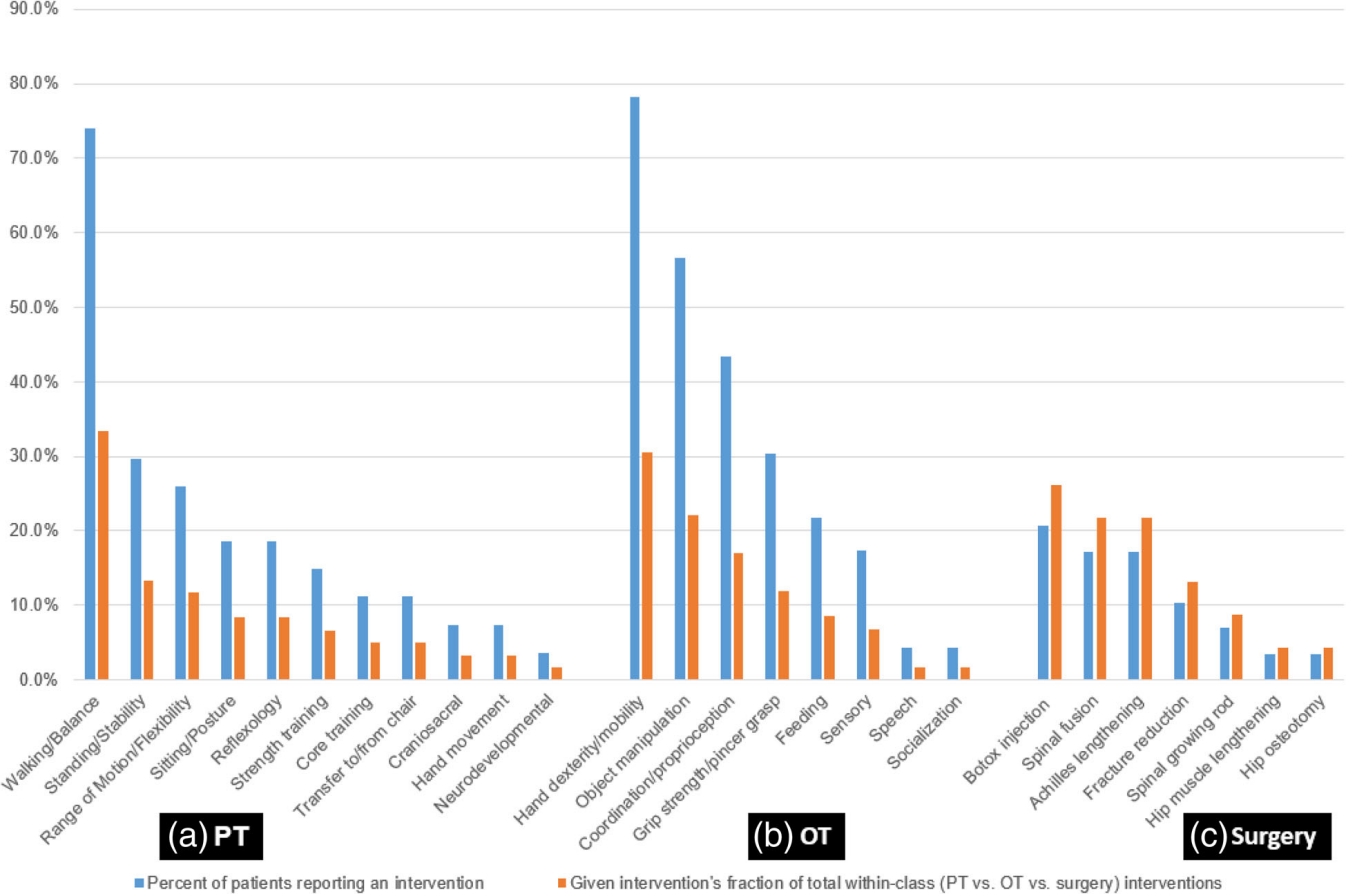
Frequency of intervention types amongst all patients and within intervention class for physical therapy (PT), occupational therapy (OT), and surgery

In addition to the use of orthoses, 27 (93.1%) and 23 (79.3%) of patients described the past or present use of physical therapy (PT) and occupational therapy (OT), respectively. For PT, patients were most commonly seen more than once a week (as was the case for 13 patients, or 48.1% of those in PT). Weekly recurrence was the next most common, reported by 5 (18.5%) patients in PT. Patients often reported multiple focus areas per session, and conducted a variety of activities in physical therapy. Walking/balance training was the most common, reported by 20 patients (74.1% of those in PT), and constituting one-third of all activities conducted in sessions (Figs. 4a). For OT, patients were most commonly seen weekly or multiple times per week (comprising of 9 patients each, together representing 78.3% of the total in OT). Patients again often reported multiple activities per session, with hand dexterity/mobility training as the most common (18 patients, or 78.3% of those in OT) (Fig. 4b).

Both males and females reported the use of orthoses at similar rates at any age (78.6% of males and 80.0% of females). More than 9 in 10 males and females older than 2 years old reported using orthoses (91.7 and 92.3%, respectively). Males more commonly used upper extremity splints (35.7% vs. 26.7% for females), whereas females were more commonly prescribed lower extremity and spinal braces (in 80.0 and 33.3% respectively, vs. 64.3 and 21.4% of males). Females were also more likely to report multiple braces (60.0% vs. 42.9% of males).

Regarding the use of PT/OT, overall utilization was similar between sexes. However, 78.6 and 50.0% of males receiving PT and OT respectively underwent therapy greater than once per week—compared with 26.7% of females for both PT and OT. Walk therapy was the most common regimen across sexes. Consistent with the increased likelihood of males to exhibit poor motor milestone development, PT regimens more often consisted of seated posture and transfer training as well as general strength training, reported by greater than one-fifth of males (versus no more than 13.3% of females reporting any of these regimens). PT sessions for females, in contrast, more commonly involved standing stability (in one-third) as well as reflexology (in 20.0%), consistent with milestone achievement patterns as well as tendencies to develop contractures. In turn, OT sessions for males disproportionately involved proprioceptive therapy, whereas for females it included grip/grasp training as well as independent feeding. Across sexes, upper extremity dexterity was the most common regimen.

### Medical therapies for musculoskeletal disorders

Thirteen patients (44.8%) were prescribed medications to support bone health. Of patients 10 years or older, 10 (76.9%) used medications for bone health. Several patients took multiple drugs. The most common prescribed medication was Vitamin D in 11 patients (84.6% of those taking medications, and 37.9% of the total), followed by calcium and bisphosphonates in 4 patients (30.7%) of those taking medications, and 13.8% of the total) each. Vitamin D constituted 57.8% of the total prescriptions, whereas calcium and bisphosphonates constituted 21.1% each.

Additionally, 4 patients (10.3% of the total) used antispasmodic agents prescribed to lower baseline muscle tone. Each of these patients used at least one different medication: one used baclofen, a second used carnitine (presumably off-label), and the third used madopar (after failing baclofen previously).

Considering sex, males were more likely to be prescribed medications than were females (57.1% vs. 46.7%, respectively). Notably, of males receiving prescriptions for bone health, they were more likely to receive prescriptions for bisphosphonates (21.4% vs. 6.7% of females), whereas females more commonly reported prescriptions for calcium (28.6% vs. 16.7% in males).

### Procedural interventions for musculoskeletal disorders

**Fig. 5:**
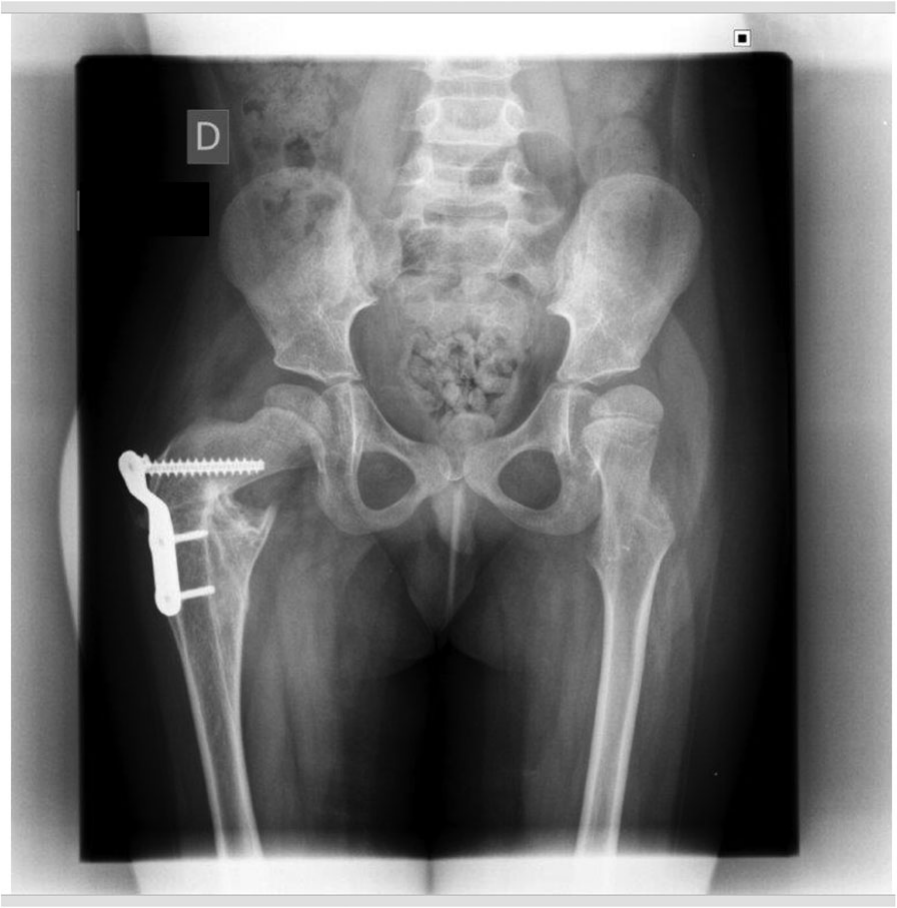
AP pelvis radiograph demonstrating another NGLY-1 deficient patient who has undergone proximal femoral osteotomy to treat neuromuscular hip dysplasia and hip subluxation

Nearly half of patients (14, or 48.3%) had an invasive procedure or surgery for a musculoskeletal disorder, and the majority of these had multiple procedures (8, or 57.1% of the procedure cohort). Of the 18 patients older than 6 years, two-thirds had at least one orthopedic procedure. The most common procedure was botulinum toxin (botox) injection for muscle spasticity, reported by 6 patients (42.9% of this cohort). Two-thirds required multiple injections, and half required injections in more than one anatomical location. Spinal fusion, Achilles tendon lengthening, and fracture reductions were the next most common (Figs. 4c, 5). Of the 18 reported fractures, 3 (16.7%) were treated with open reduction.

Both sexes underwent procedures at similar rates (50.0% of males and 46.7% of females), but the distribution of procedures differed between them. Males were more likely to undergo Botox injections, spinal fusion, and hip muscle/bone corrections. In contrast, females disproportionately received Achilles tendon lengthening and open fracture reduction.

## Discussion

Our findings represent the first report detailing the orthopedic manifestations, gross motor development, and musculoskeletal treatments of patients with NGLY1 deficiency. This is the largest study cohort compiled for the disease to date. These findings provide information upon which physicians can better counsel patients with NGLY1 deficiency on possible orthopaedic issues.

For example, fracture rates higher than index populations coupled with high rates of repeat fracture can be used to inform individualized prophylactic measures (such as fall prevention measures within the household, safe lifting and transfer techniques, and lower extremity bracing). High frequency of orthosis, OT, and PT utilization, as well as the high observed rate of motor skills regression, can facilitate parental expectations regarding disease natural history. The documented range of medications can guide individualized choices to muscle tone management (oral medications, botox) and maximize bone health (such as preferential use of Vitamin D, calcium, and bisphosphonates). The observation that half of all patients underwent invasive procedures inform communications about expectations, planning, and management in specific cases—including the potential for some NGLY1 deficiency patients to develop severe spinal and hip deformities that may need major surgery. Stratification of these analyses by sex revealed distinctive patterns across disease natural history and management course, which can likewise guide expectations as it relates to patient care over the long term.

The musculoskeletal disorders documented here in NGLY1 deficiency are similar to those seen in other upper motor neuron disorders such as cerebral palsy (CP), but the clinical course of motor skills regression parallels other neurodegenerative disorders, such as Rett syndrome [23]. Abnormal muscle tone, poor trunk and extremity control, and altered balance lead to limited weight bearing and ambulation across these conditions. Similarly, contractures, scoliosis and hip subluxation/dislocations are seen in NGLY1 deficient individuals similar to those with CP, and the principles of treatment are analogous. Also akin to CP patients, significant spinal deformity impairs sitting ability, and hip contractures and dislocations can lead to pain and difficulty with perineal hygiene, in addition to impaired sitting. Finally, poor bone health and limited weight-bearing combined with altered balance and coordination leading to falls predispose these populations to increased fracture rates [14]. Thus, information on clinical course for NGLY1 deficiency may be informative to physicians treating patients with these conditions as well.

The methodology of this study may be applied to other rare diseases, as partnership with disease advocacy organizations presents an opportunity to balance “bottom up” origins of research (initiated by scientists) with “top down” ones (solicited by patient families, through clinicians) [24]. This approach fills voids in experimental efforts (particularly important for rare disorders) and ensures inclusion of patient-relevant outcomes (PROs) in study design [25]. Including PROs in study design facilitates informed shared decision-making fundamental to *patient-centered care* [26]. It also prevents misalignment between research questions pursued by investigator-initiated studies, and answers sought by patients [27, 28].

It has been noted that investigator-initiated studies tend to pursue pharmacologic therapies (seeking future cures), while affected families tend to prioritize non-pharmacologic treatments (in lieu of current cures). One study addressing this deemed “research priorities gap” found that while drug therapies were prioritized in only 18% of responses by patients, they accounted for up to 86% of trials initiated by investigators [29]. Simultaneously, fewer than 3% of investigator-initiated studies addressed non-pharmacologic therapies [29]. This is a considerable mismatch not only from the standpoint of patient autonomy, but also because of the risk-benefit ratio: 97% of orphan drug therapies cause adverse events, yet less than one-fifth demonstrate clinical improvement [30]. Similar patterns exist in research addressing NGLY1 deficiency, as the majority of therapeutic research has surrounded gene or biologic interventions [12, 16, 31]. We could not find any studies addressing non-pharmacologic therapy for the disease. While families and investigators are vested in research to find cures, research addressing the consequences of the disorder to improve function, mobility and prevent adverse sequelae is also needed.

Finally, the partnership exemplified by this study may become increasingly relevant with the advent of personalized medicine. Accessibility of genome sequencing methods may reveal greater genotypic variability than previously understood, leading to the (i) discovery (ii) subcategorization or (iii) reclassification of diseases [24, 32]. Common diseases may become stratified into successively smaller cohorts, each with distinctive clinical courses demanding distinctive treatments (what has been deemed *salami-slicing*) [33]. In this context, alliances between physicians and “patient-driven information economies” driven by *patient informaticians* may present the best opportunities for clinical care in rare and common conditions alike [34, 35].

There are several limitations to our study. The first relates to timing of survey completion relative to reported elements of the natural history and management therein. Given that the survey relied upon self-reporting by patients with varying time delay from aspects of their clinical history, the recorded values are vulnerable to recall bias. It is possible that, as with all patient-centric survey tools, all aspects of the clinical history were not captured, and those that were captured are imperfectly accurate. Additionally, since symptom, diagnostic, and procedural classification systems vary between countries, inconsistencies in clinical histories may exist that were not reflected in the recorded data.

## Conclusions

In sum, orthopaedic manifestations are common in patients with NGLY1 deficiency and clinical interventions are frequently required. To date, these manifestations have been incompletely described and practices used for clinical management have not been fully characterized. In this study, we have comprehensively described the orthopaedic natural history and catalogued the current standards of care in clinical practice. These findings can facilitate diagnosis, inform prognosis, and guide treatment recommendations in an evidence-based manner for patients with orthopaedic manifestations related to NGLY1 deficiency. Additionally, the design of our study, through partnership with an international disease-specific advocacy organization and premised on patient-centric clinical questions, offers a research methodology that may be generalizable to other rare and/or common diseases in the future.

## Data Availability

The datasets used and/or analyzed during the current study are available from the corresponding author on reasonable request.
